# Supplementary material for: Extreme diversity of 12 cations in folding ALS-linked hSOD1 unveils novel hSOD1-dependent mechanisms for Fe2+/Cu2+-induced cytotoxicity
Source: Sci Rep. 2023 Nov 14;13:19868. doi: 10.1038/s41598-023-47338-8 (PMC10645853; doi:10.1038/s41598-023-47338-8)
Supplement: Supplementary file 1 — Supplementary Information. [file 41598_2023_47338_MOESM1_ESM.docx]

**Supplementary Materials**


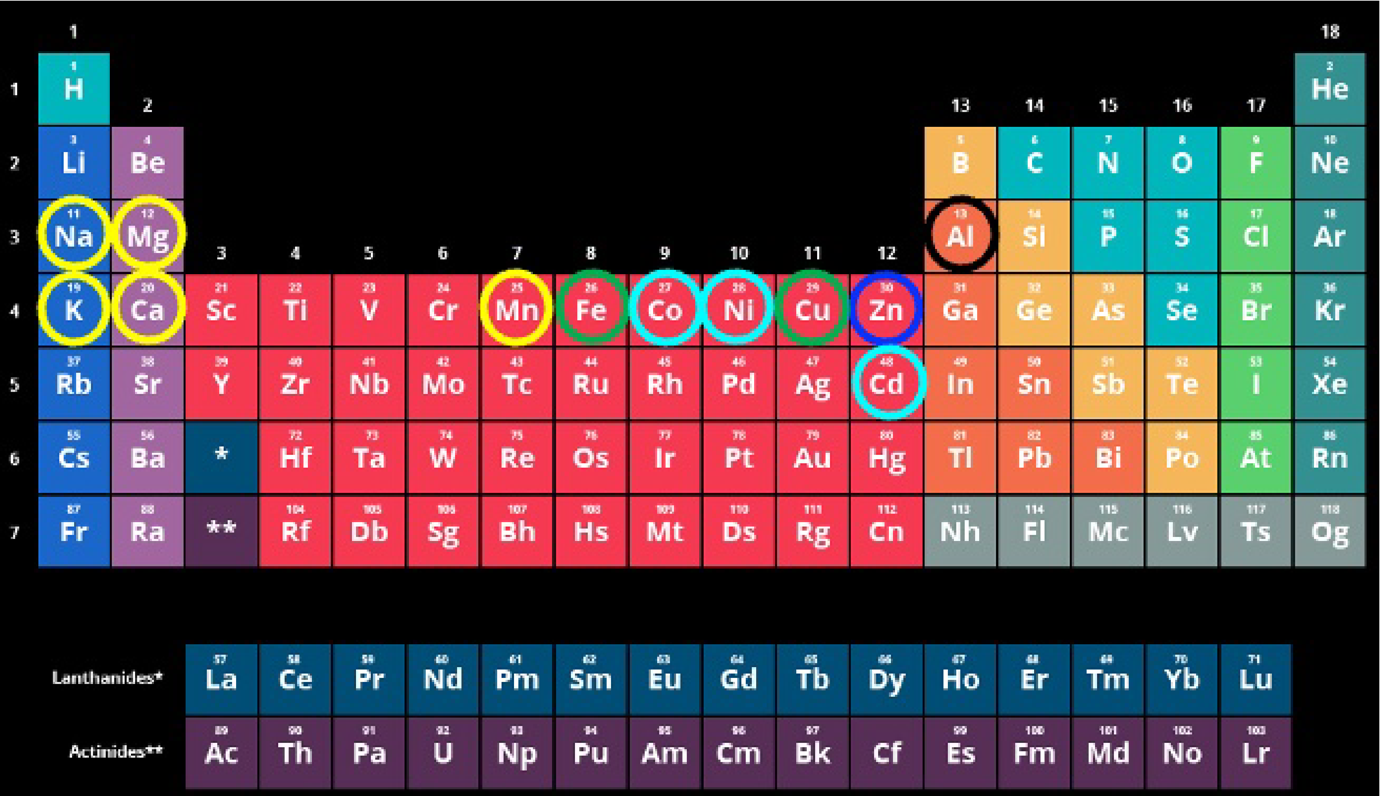


**Fig. S1. Selection of 12 cations.**

The periodic table of the elements to indicate the location of 12 metal cations selected for the present study.


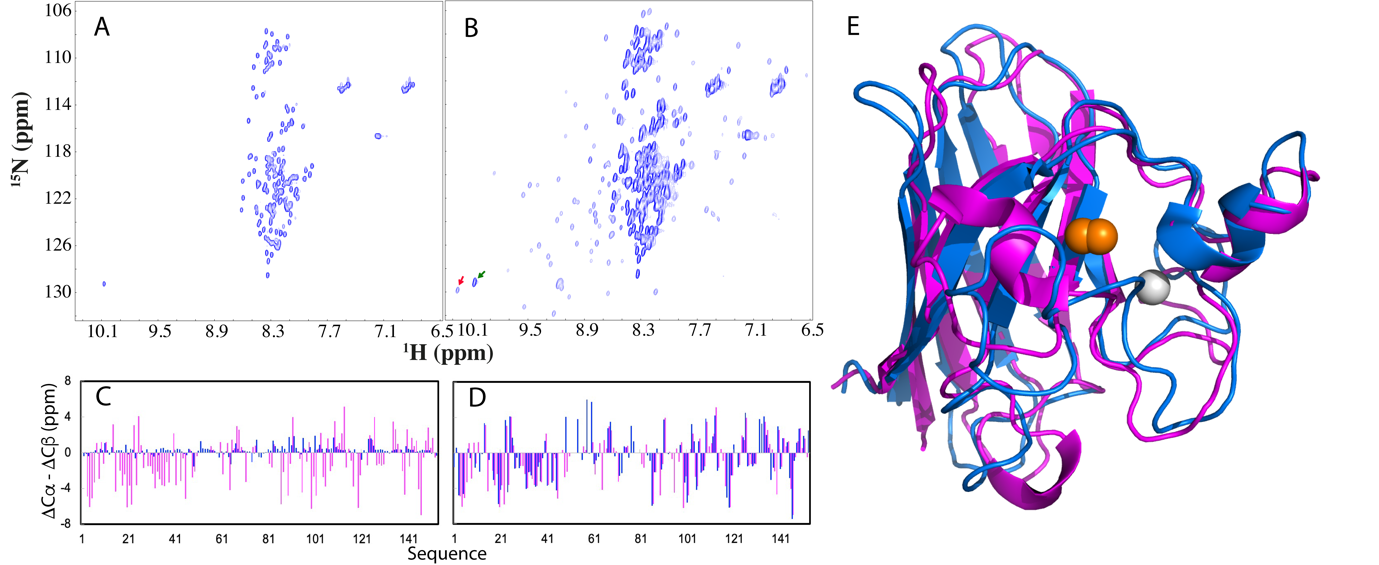


**Fig. S2. Zn^2+^-induced folding of nascent hSOD1.**

HSQC spectra of nascent hSOD1 in the absence (A) and in the presence of Zn^2+^ at a molar ratio of 1:20 (B). (C) Residue specific (ΔCα–ΔCβ) chemical shifts of nascent hSOD1 (blue) and in the presence of Zn^2+^ at a molar ratio of 1:20 (purple). (D) Residue specific (ΔCα–ΔCβ) chemical shifts of the Zn^2+^-induced hSOD1 and those of C6A/C111S (blue) (BMRB Entry of 6821). (E) Superimposition of the NMR structure of the super-stable pseudo-WT hSOD1 C6A/C111S (PDB ID of 2AF2) without the disulfide bridge and copper (blue) and crystal structure (PDB ID of 2C9V) of mature hSOD1 (purple). Zinc ion is in grey sphere and copper in orange sphere.


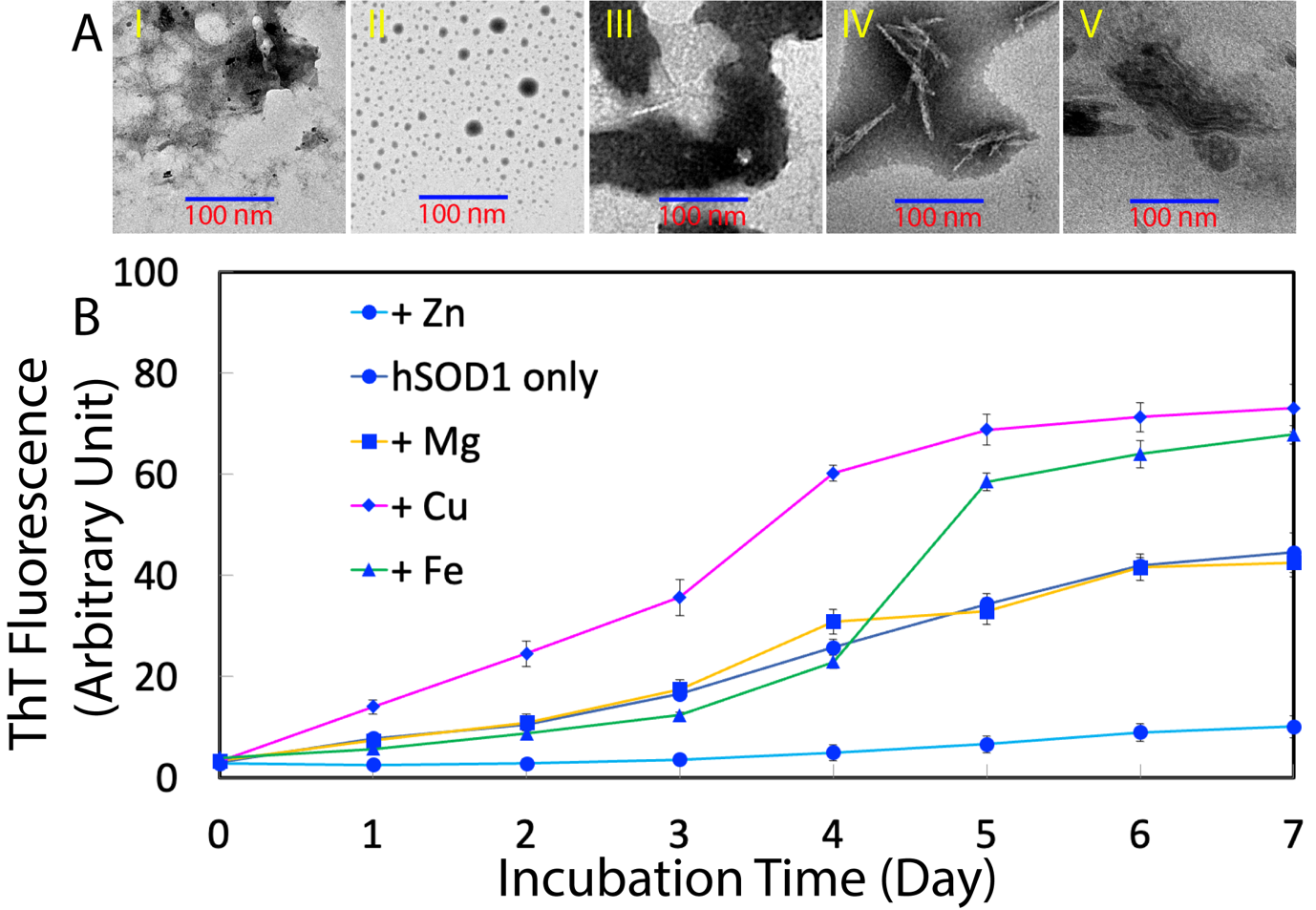


**Fig. S3. Characterization of aggregation and amyloid formation of the hSOD1 samples.**

(A) EM images of the hSOD1 samples in the absence (I) and in the presence of Zn^2+^ (II), Mg^2+^ (III), Cu^2+^ (IV) and Fe^2+^ (V) at molar ratios of 1:20 after 7 days of the incubation. (B) ThT-binding induced fluorescence at 486 nm of the different hSOD1 samples during the incubation.


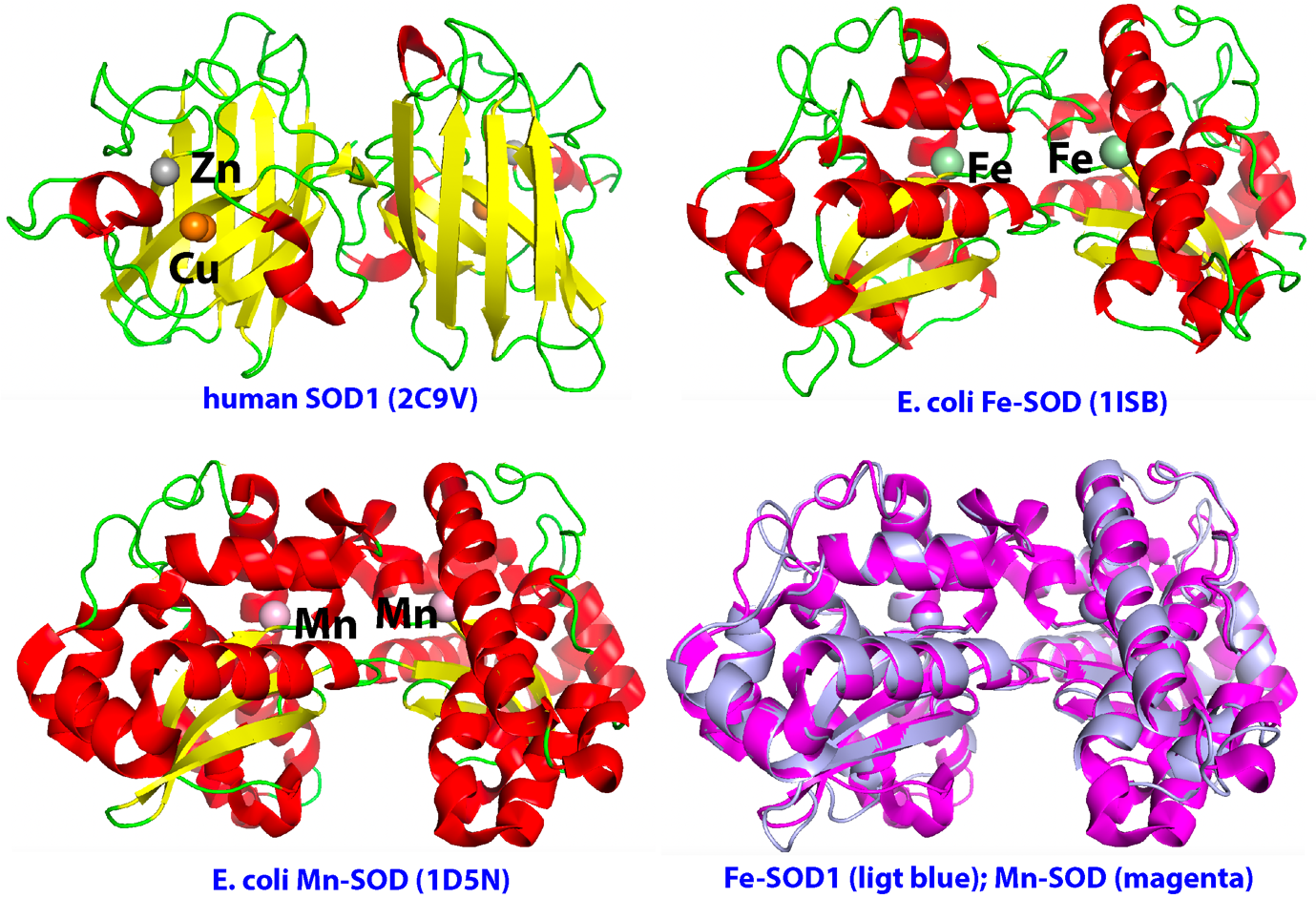


**Fig. S4. Three-dimensional structures of human CuZn-superoxide dismutase 1 (hSOD1), as well as *E. coli* Fe-SOD and Mn-SOD.**
